# Supplementary material for: Unravelling reference bias in ancient DNA datasets
Source: Bioinformatics. 2024 Jul 3;40(7):btae436. doi: 10.1093/bioinformatics/btae436 (PMC11254355; doi:10.1093/bioinformatics/btae436)
Supplement: btae436_Supplementary_Data [file btae436_supplementary_data.zip › S1-S4_data_plots.zip!/S1-S4_data/rhino_sim_plots.zip!/rhino_sim_plots/local_bowtie_rhino_div12_1end_by_MQ.pdf]

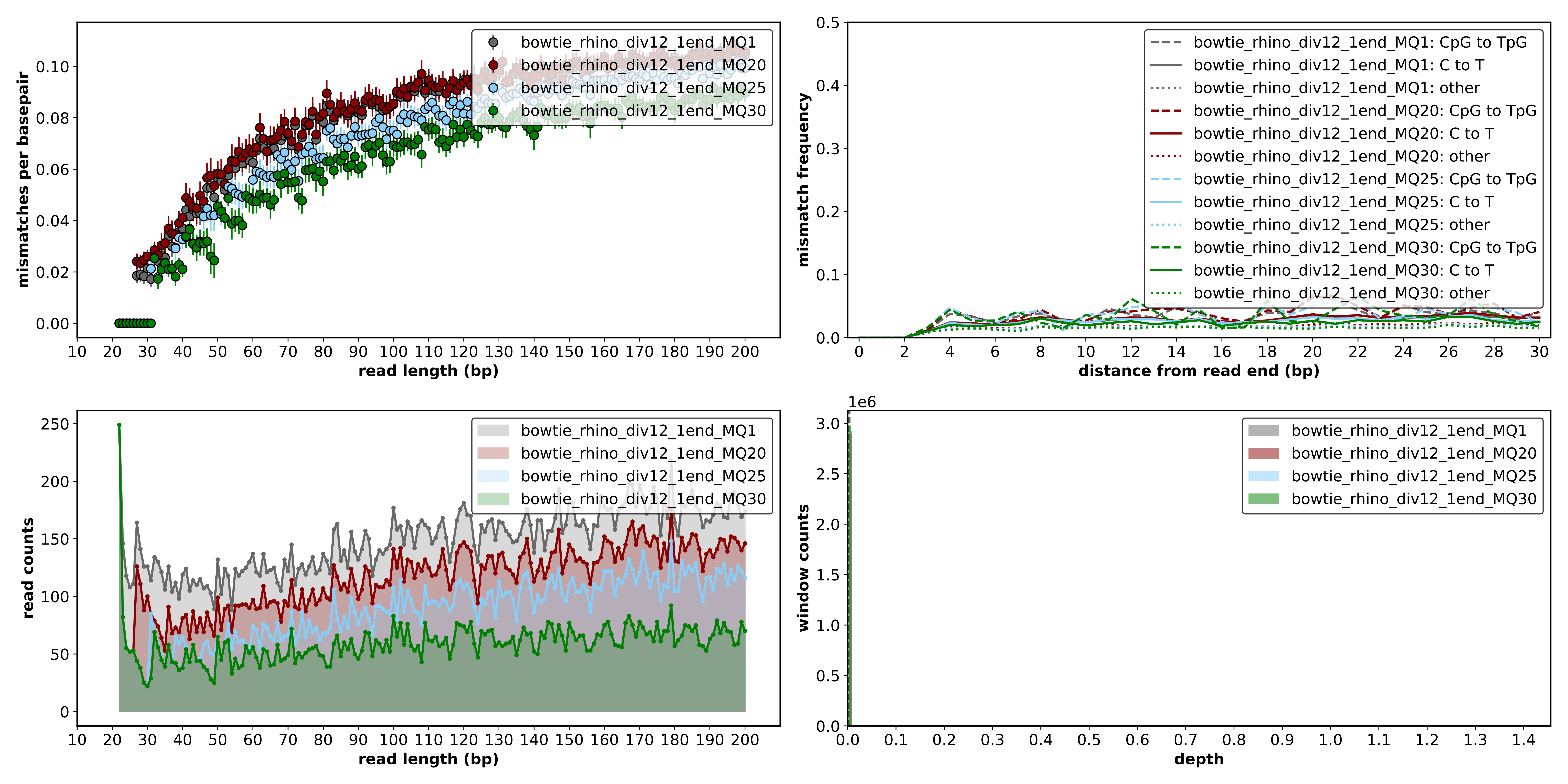

mismatches per basepair
read length (bp)
bowtie_rhino_div12_1end_MQ1
bowtie_rhino_div12_1end_MQ20
bowtie_rhino_div12_1end_MQ25
bowtie_rhino_div12_1end_MQ30
mismatch frequency
distance from read end (bp)
bowtie_rhino_div12_1end_MQ1: CpG to TpG
bowtie_rhino_div12_1end_MQ1: C to T
bowtie_rhino_div12_1end_MQ1: other
bowtie_rhino_div12_1end_MQ20: CpG to TpG
bowtie_rhino_div12_1end_MQ20: C to T
bowtie_rhino_div12_1end_MQ20: other
bowtie_rhino_div12_1end_MQ25: CpG to TpG
bowtie_rhino_div12_1end_MQ25: C to T
bowtie_rhino_div12_1end_MQ25: other
bowtie_rhino_div12_1end_MQ30: CpG to TpG
bowtie_rhino_div12_1end_MQ30: C to T
bowtie_rhino_div12_1end_MQ30: other
read counts
read length (bp)
bowtie_rhino_div12_1end_MQ1
bowtie_rhino_div12_1end_MQ20
bowtie_rhino_div12_1end_MQ25
bowtie_rhino_div12_1end_MQ30
1e6
window counts
depth
bowtie_rhino_div12_1end_MQ1
bowtie_rhino_div12_1end_MQ20
bowtie_rhino_div12_1end_MQ25
bowtie_rhino_div12_1end_MQ30
